# Antidepressant Use and Bone Health: Evidence From Mendelian Randomisation

**DOI:** 10.62641/aep.v54i2.2164

**Published:** 2026-04-15

**Authors:** Yinqing Huang, Lingzhi Lin, Mian Lin, Xiuxiu Ye

**Affiliations:** ^1^Department of Medicine, The Affiliated Kangning Hospital of Wenzhou Medical University, 325000 Wenzhou, Zhejiang, China; ^2^Department of Orthopedics, The Third Affiliated Hospital of Wenzhou Medical University, 325000 Wenzhou, Zhejiang, China

**Keywords:** antidepressants, osteoporosis, fractures, Mendelian randomsation, genome-wide association study

## Abstract

**Background:**

Antidepressant use has been associated with adverse skeletal outcomes in observational studies, but whether this association is causal remains unclear due to potential confounding factors. This study employed Mendelian randomisation (MR) to systematically evaluate the causal associations between genetically predicted antidepressant use and the risks of osteoporosis and fractures at multiple anatomical sites.

**Methods:**

Using publicly available genome-wide association studies (GWAS) datasets, we defined genetically predicted antidepressant use as the exposure and osteoporosis and fractures (spine, leg, and wrist) as outcomes. Genome-wide significant single nucleotide polymorphisms (SNPs) were selected as instrumental variables following dataset harmonisation. The inverse-variance weighted (IVW) method was used as the primary MR approach, supplemented by MR-Egger regression, weighted median, and weighted/simple mode methods. Heterogeneity tests, funnel plots, and leave-one-out sensitivity analyses were performed to assess the robustness and consistency of the results.

**Results:**

MR analysis demonstrated significant positive causal associations between antidepressant use and osteoporosis across two independent datasets (ukb-a-87 and ukb-b-12141), with IVW odds ratios of 1.0035 and 1.0016, respectively. Genetically proxied antidepressant use showed significant causal effects on fractures at all examined sites, with wrist fractures displaying the strongest association (IVW: odds ratio (OR) = 1.0027, *p* = 0.0041). Effect directions remained consistent across multiple MR methods, with no significant heterogeneity detected. Leave-one-out analyses confirmed no single SNP disproportionately influenced the results.

**Conclusion:**

This MR study provides evidence that antidepressant use may directly influence bone metabolism and increase fracture susceptibility, particularly at the wrist. These findings highlight the importance of bone health monitoring in patients receiving antidepressant therapy, especially those at elevated fracture risk. Further mechanistic studies and longitudinal validation are warranted.

## Introduction

Antidepressant medications are widely used in clinical practice to treat depression and anxiety disorders. Moreover, researchers have investigated their potential applications in conditions such as cancer and arthritis [1, 2, 3, 4]. However, the side effects of antidepressants are garnering increasing attention, including gastrointestinal symptoms, hepatotoxicity, cardiovascular disturbances, genitourinary issues, sexual dysfunction, and hyponatremia [5, 6, 7, 8].

Osteoporosis, characterised by decreased bone mineral density (BMD) and an elevated risk of fractures, poses a substantial public health concern, especially among the aging population [9]. Numerous factors influence bone health, including age, sex, hormonal imbalances, and lifestyle factors such as physical activity and diet [10]. Among these factors, medication use, particularly of drugs targeting the central nervous system, has garnered increasing recognition for their potential impact on bone metabolism [11].

While previous studies have established an association between certain psychotropic medications and osteoporosis [12, 13, 14], the role of antidepressants remains under investigation. The relationship between antidepressant use, osteoporosis, and fracture risk remains inconclusive. A population-based study suggests that antidepressant use is correlated with an increased likelihood of being prescribed osteoporosis medications later in life, indicating a potential link between long-term antidepressant use and declining bone health [15]. A study reported that antidepressants are associated with decreased BMD and a heightened risk of fractures, although earlier research did not consistently categorise antidepressants within this group [16]. Recent systematic reviews and meta-analyses provide evidence supporting the association between antidepressant use, particularly serotonergic antidepressants, and an increased risk of both osteoporosis and fractures [17, 18, 19, 20]. Given these inconsistencies, traditional observational studies are insufficient to establish whether the relationship between antidepressant use and bone outcomes is causal.

Mendelian randomisation (MR) is a statistical method employed to examine the causal relationship between exposure factors and outcomes, utilising genetic variants as instrumental variables [21]. First introduced by George Davey Smith and colleagues in 2003 [22], MR provides insights into the environmental determinants of disease and offers a formal research framework. By leveraging genetic data, MR can minimise bias akin to randomised controlled trials and has been widely used to investigate causal relationships between exposure factors and outcomes.

Despite growing evidence linking antidepressant use to adverse bone outcomes in observational studies, the causal nature of this relationship remains uncertain due to residual confounding, reverse causation, and the challenge of disentangling medication effects from the underlying psychiatric conditions being treated. Traditional observational approaches cannot definitively establish whether antidepressants directly impair bone health or whether the association reflects confounding by indication, comorbidities, or lifestyle factors associated with depression. This critical knowledge gap has important clinical implications given the widespread use of antidepressants in populations at elevated fracture risk. Therefore, this study employs a two-sample MR design to overcome these limitations and rigorously examine the potential causal relationship between genetically predicted antidepressant use and bone health outcomes, including osteoporosis and fractures at multiple skeletal sites. By leveraging genetic variants as unconfounded proxies for antidepressant exposure and integrating large-scale GWAS datasets with multiple analytical methods, we aim to provide causal evidence to inform clinical decision-making regarding bone health monitoring in patients receiving antidepressant therapy.

## Materials and Methods

### Study Design

**Fig. 1.**
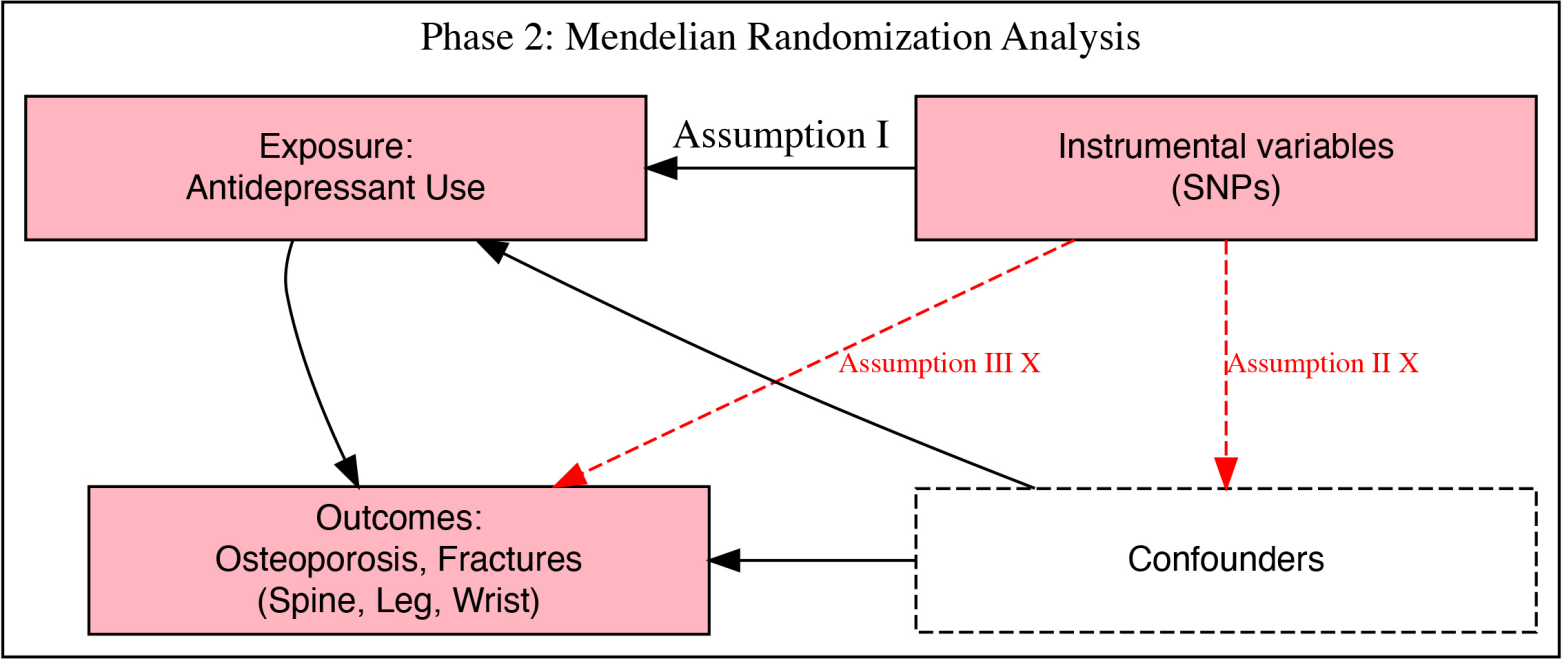
**Overview of assumptions for Mendelian randomisation (MR)**. Assumption I (Relevance Assumption): single nucleotide polymorphisms (SNPs) (instrumental variables) must be associated with the exposure (antidepressant use). Assumption II (Independence Assumption): SNPs must not be associated with any confounders. Assumption III (Exclusion Restriction Assumption): SNPs should affect the outcomes only through their effect on the exposure (antidepressant use). The red “X” indicates no violation of this assumption.

Within the framework of MR analysis, this study conducted a two-sample MR analysis using published genome-wide association studies (GWAS) data to assess the causal association between antidepressant use and the risk of osteoporosis and fractures (leg, spine, and wrist). In this study, antidepressant use was defined as the exposure, and osteoporosis, spinal fractures, leg fractures, and wrist fractures were taken as outcomes, as detailed below (Fig. 1).

### Sources of MR

We employed a two-sample MR approach using publicly available GWAS summary data from the Integrative Epidemiology Unit Open GWAS Project (https://gwas.mrcieu.ac.uk). The exposure variable was genetically proxied antidepressant use, derived from a GWAS by Sakaue et al. [23]. The outcome GWAS summary statistics for spine fracture (ukb-b-873), Wrist fracture (ukb-b-9571), Leg fracture (ukb-b-3798) and self-reported osteoporosis (ukb-a-87 and ukb-b-12141) were obtained from the UK Biobank via the IEU OpenGWAS database [24]. These datasets were generated using the MRC-IEU GWAS pipeline based on UK Biobank participants of European ancestry. The specific information on the outcomes and exposure factors included in this study is presented in Table 1. The F-statistics for all instrumental variables substantially exceeded the conventional threshold of 10, indicating a low likelihood of weak instrument bias.

**Table 1.** **Detailed information on exposure and outcome factor**.

Exposure and outcomes	Dataset	Trait	Year	Population	Sample size	Cases (*N*)	Controls (*N*)	Number of SNPs	F	R^2^
Antidepressant use	ebi-a-GCST90018998	Medication use (antidepressants)	2021	European	304,162	33,757	270,405	14,256,555	—	—
Fractured Spine	ukb-b-873	Fractured bone site(s): Spine	2018	European	460,340	1036	459,304	9,851,867	14.4312	0.0019
Fractured Wrist	ukb-b-9571	Fractured bone site(s): Wrist	2018	European	460,340	9113	451,227	9,851,867	19.2651	0.0044
Fractured Leg	ukb-b-3798	Fractured bone site(s): Leg	2018	European	460,340	2988	457,352	9,851,867	19.6701	0.0032
Osteoporosis	ukb-a-87	Non-cancer illness code self-reported: osteoporosis	2017	European	337,159	5266	331,893	10,894,596	19.5835	0.0041
Osteoporosis	ukb-b-12141	Non-cancer illness code, self-reported: osteoporosis	2018	European	462,933	7547	455,386	9,851,867	19.3178	0.0036

### Statistical Analysis

For the MR analysis, we explored the causal relationship between antidepressant use and the risk of osteoporosis and fractures (specifically leg, spine, and wrist). Instrumental variables (IVs) were selected from genome-wide significant SNPs with *p*-values less than 1 × 10^-6^ [25]. Linkage disequilibrium thresholds were set at r^2^< 0.001 and a genetic distance of 10 megabases to ensure independence among instruments. Harmonisation of exposure and outcome datasets was conducted to consistently align effect alleles. The strength of instrumental variables was assessed using the F-statistic, calculated as F = R^2^ (N – K – 1) / K(1 – R^2^), where R^2^ represents the proportion of variance in the exposure explained by the genetic instruments, N is the sample size, and K is the number of instruments. An F-statistic >10 indicates strong instruments and minimal weak instrument bias.

The primary MR analysis used the inverse-variance weighting (IVW) method under the assumption of no horizontal pleiotropy. Sensitivity analyses included the leave-one-out method to assess the influence of individual SNPs. Pleiotropy was evaluated using the MR-Egger intercept test; a non-zero intercept suggests directional pleiotropy. Heterogeneity among SNPs was assessed using Cochran’s Q statistic. The strength of the genetic instruments was tested using F-statistics, with values greater than 10 indicating strong instruments.

Results from the MR analyses are reported as odds ratios (ORs) with 95% confidence intervals (CIs) derived from the IVW method, supplemented by MR-Egger regression results and heterogeneity assessments. All analyses were conducted using R software (version 4.4.1; R Foundation for Statistical Computing; https://www.r-project.org). MR analyses were performed using the TwoSampleMR package (version 0.6.7; MRC Integrative Epidemiology Unit; https://mrcieu.github.io/TwoSampleMR/). Data manipulation and visualisation were conducted using ggplot2 (3.5.2; R Foundation for Statistical Computing; https://cran.r-project.org/package=ggplot2) and VariantAnnotation (1.50.0Bioconductor Project; https://bioconductor.org/packages/VariantAnnotation/).

Given the exploratory nature of this study and the biological relatedness of the outcomes (all representing aspects of bone health), we did not apply strict Bonferroni correction for multiple testing across the five outcomes. However, we acknowledge that multiple comparisons increase the probability of type I error. To address this concern, we adopted a conservative approach by: (1) requiring *p* < 0.05 for statistical significance in the primary IVW analysis, (2) demanding consistency across multiple MR methods, and (3) conducting comprehensive sensitivity analyses. Results that showed statistical significance, methodological consistency, and biological plausibility were considered robust findings.

## Results

### The Causal Relationship Between Antidepressant Use and Osteoporosis

**Fig. 2.**
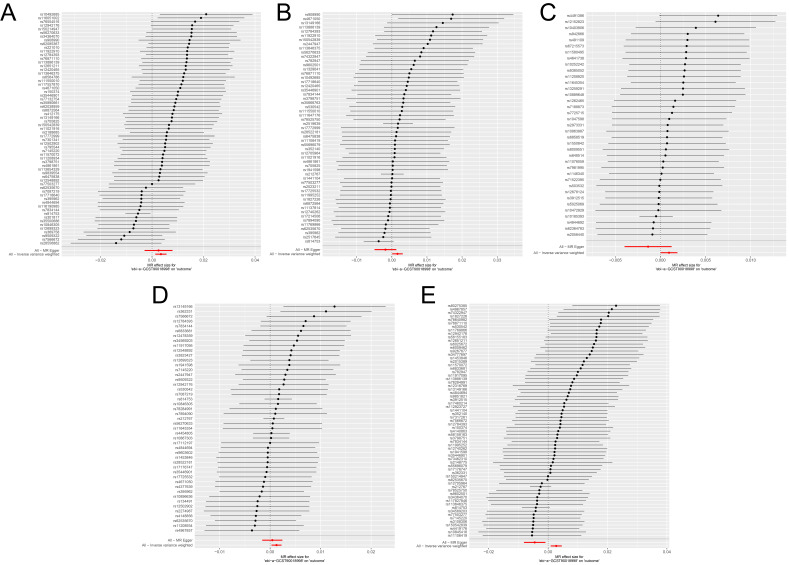
**Forest plot of MR analysis results**. (A–E) Forest plot showing IVW and MR-Egger analysis results of causal associations between antidepressant use and osteoporosis [ukb-a-87 (A) and ukb-b-12141 (B)], spine fractures [ukb-b-873 (C)], leg fractures [ukb-b-3798 (D)], and wrist fractures [ukb-b-9571 (E)].

The IVW method revealed significant causal associations between antidepressant use and both osteoporosis outcomes (Table 2). For ukb-a-87, the IVW method yielded an OR of 1.0035 (95% CI: 1.0013–1.0056, *p* = 0.0019) (Fig. 2A). For ukb-b-12141, the IVW method showed an OR of 1.0016 (95% CI: 1.0001–1.0031, *p* = 0.0354) (Fig. 2B). These findings demonstrate statistically significant positive causal associations between antidepressant use and osteoporosis risk across two independent datasets.

**Table 2.** **Statistical data of MR analysis**.

Outcome	Method	SNPs	*p*-value	Odds ratio (95% CI)
ukb-a-87	MR Egger	63	0.3624	1.0025 (0.9971, 1.0080)
	WM	63	0.0076	1.0043 (1.0011, 1.0074)
	IVW	63	0.0019	1.0035 (1.0013, 1.0056)
	SM	63	0.0298	1.0105 (1.0012, 1.0199)
	WMo	63	0.0857	0.9947 (0.9888, 1.0007)
ukb-b-12141	MR Egger	55	0.2558	0.9982 (0.9951, 1.0013)
	WM	55	0.8659	1.0002 (0.9977, 1.0027)
	IVW	55	0.0354	1.0016 (1.0001, 1.0031)
	SM	55	0.9119	1.0003 (0.9957, 1.0049)
	WMo	55	0.6914	1.0005 (0.9981, 1.0029)
ukb-b-873	MR Egger	37	0.3087	0.9986 (0.9960, 1.0012)
	WM	37	0.6871	1.0003 (0.9989, 1.0017)
	IVW	37	0.0470	1.0009 (1.0000, 1.0019)
	SM	37	0.8671	1.0002 (0.9975, 1.0030)
	WMo	37	0.7801	0.9998 (0.9982, 1.0014)
ukb-b-3798	MR Egger	47	0.6703	1.0004 (0.9984, 1.0024)
	WM	47	0.3488	1.0007 (0.9992, 1.0023)
	IVW	47	0.0127	1.0013 (1.0003, 1.0023)
	SM	47	0.8926	0.9998 (0.9973, 1.0024)
	WMo	47	0.3443	1.0007 (0.9993, 1.0021)
ukb-b-9571	MR Egger	67	0.0170	0.9955 (0.9919, 0.9991)
	WM	67	0.8741	0.9998 (0.9972, 1.0024)
	IVW	67	0.0041	1.0027 (1.0009, 1.0046)
	SM	67	0.4359	1.0031 (0.9954, 1.0109)
	WMo	67	0.0534	0.9973 (0.9946, 1.0000)

Abbreviations: CI, confidence interval; IVW, Inverse Variance Weighted; WM, Weighted Median; MR Egger, Mendelian Randomisation Egger; SM, simple mode; WMo, weighted mode. Exposure: Antidepressant use.

### The Causal Relationship Between Antidepressant Use and Fracture

Significant causal associations were identified between genetically proxied antidepressant use and fractures at all three anatomical sites examined (Table 2). For spine fractures (ukb-b-873), the IVW method reported an OR of 1.0009 (95% CI: 1.0000–1.0019, *p* = 0.0470) (Fig. 2C). The IVW analysis of leg fractures (ukb-b-3798) yielded an OR of 1.0013 (95% CI: 1.0003–1.0023, *p* = 0.0127) (Fig. 2D). Most notably, wrist fractures (ukb-b-9571) demonstrated the strongest association among fracture outcomes, with the IVW method showing an OR of 1.0027 (95% CI: 1.0009–1.0046, *p* = 0.0041) (Fig. 2E). These findings highlight site-specific differences in fracture susceptibility associated with antidepressant use.

### Comparison of Multiple MR Methods

**Fig. 3.**
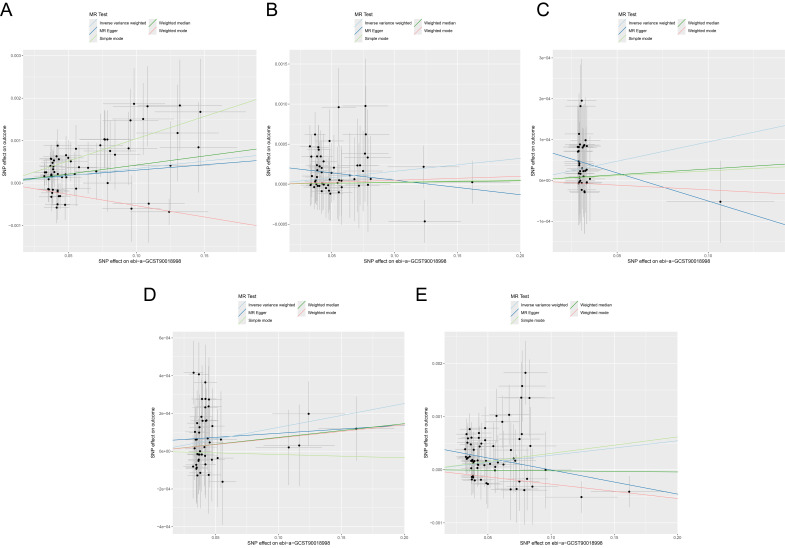
**Scatter plots of MR analysis results**. (A–E) Scatter plots showing MR analysis results of causal associations between antidepressant use and osteoporosis [ukb-a-87 (A) and ukb-b-12141 (B)], spine fractures [ukb-b-873 (C)], leg fractures [ukb-b-3798 (D)], and wrist fractures [ukb-b-9571 (E)].

Multiple MR approaches were employed to assess causal relationships, with results visualised through scatter plots (Fig. 3A–E). These included MR-Egger, weighted median (WM), IVW, simple mode (SM), and weighted mode (WMo) methods across all five outcomes. Regarding osteoporosis (ukb-a-87), positive causal effects were observed across all methods with the exception of WMo (Fig. 3A). A similar pattern of directional consistency emerged for osteoporosis (ukb-b-12141) (Fig. 3B), spine fractures (ukb-b-873) (Fig. 3C), and leg fractures (ukb-b-3798) (Fig. 3D), where most methods aligned in their effect directions. The results for wrist fractures (ukb-b-9571) presented greater heterogeneity (Fig. 3E). While MR-Egger and WMo indicated negative effects, the IVW, WM, and SM methods converged on positive associations. Notably, despite these methodological variations in certain outcomes, the primary IVW analysis yielded consistently significant positive causal associations across all five bone health outcomes. This consistent directionality of the IVW estimates, coupled with general agreement among supplementary methods, substantiates the robustness and reliability of the identified causal relationship between antidepressant use and elevated bone health risks.

### Sensitivity Analyses

**Fig. 4.**
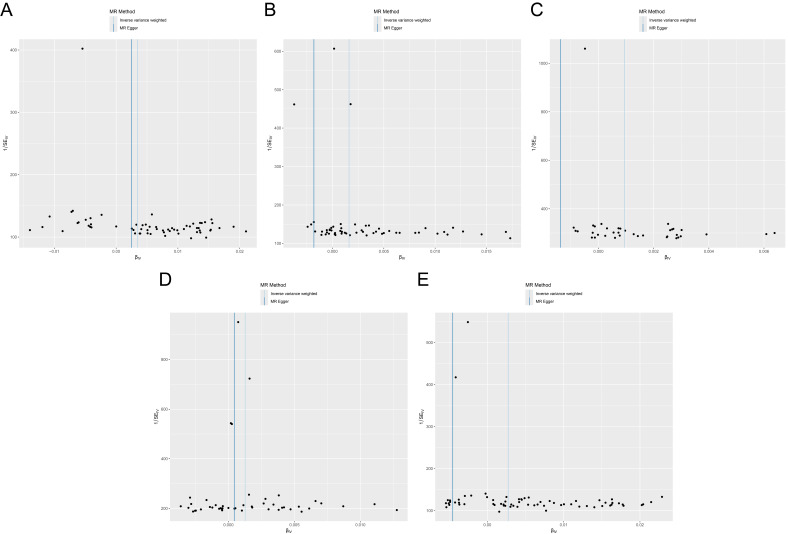
**Funnel plots of MR analysis results**. (A–E) Funnel plots showing MR analysis results of causal associations between antidepressant use and osteoporosis [ukb-a-87 (A) and ukb-b-12141 (B)], spine fractures [ukb-b-873 (C)], leg fractures [ukb-b-3798 (D)], and wrist fractures [ukb-b-9571 (E)].

**Fig. 5.**
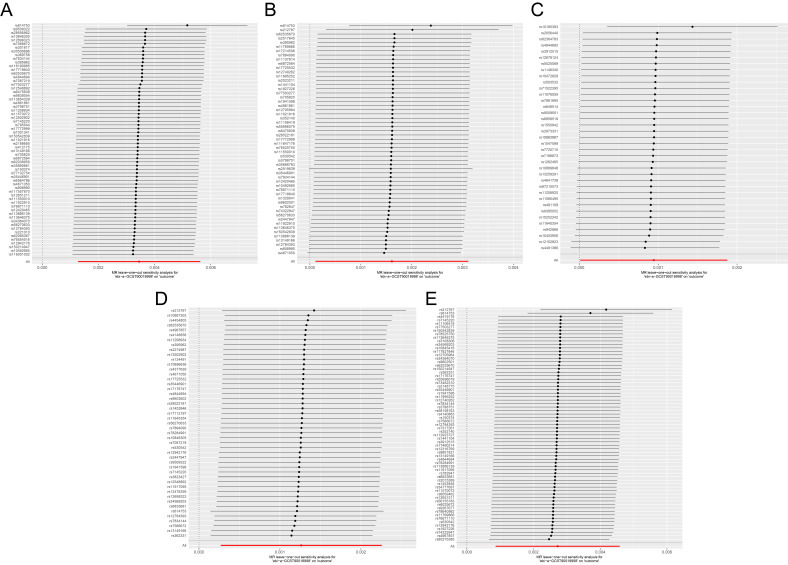
**Leave-one-out sensitivity analysis**. (A–E) Leave-one-out sensitivity analysis showing MR analysis results of causal associations between antidepressant use and osteoporosis [ukb-a-87 (A) and ukb-b-12141 (B)], spine fractures [ukb-b-873 (C)], leg fractures [ukb-b-3798 (D)], and wrist fractures [ukb-b-9571 (E)].

Heterogeneity tests using Cochran’s Q statistic indicated no significant heterogeneity among the genetic instruments for any of the five outcomes (Table 3). For osteoporosis (ukb-a-87 and ukb-b-12141), the IVW Q-test *p* values were 0.1058 and 0.9943, respectively. The corresponding *p* values were 0.9998 for spine fractures (ukb-b-873), 0.9808 for leg fractures (ukb-b-3798), and 0.0809 for wrist fractures (ukb-b-9571), suggesting consistent effect estimates across SNPs without evidence of substantial heterogeneity. Funnel plots showed symmetrical distributions of SNP effect estimates (β_IV_) around the IVW estimate line, indicating no obvious evidence of directional pleiotropy (Fig. 4A–E). Leave-one-out analyses demonstrated that the causal effect estimates and confidence intervals remained relatively stable when sequentially excluding any single SNP (Fig. 5A–E). This finding indicates that our MR analysis results are not driven by any individual SNP contribution, thereby further supporting the reliability of the causal relationship between antidepressant use and the risk of osteoporosis and fractures across multiple anatomical sites.

**Table 3.** **Heterogeneity test**.

Exposure	Outcome	Method	Q	df	*p* value
Antidepressant use	ukb-a-87	MR Egger	76.0570	61	0.0928
Antidepressant use	ukb-a-87	IVW	76.2137	62	0.1058
Antidepressant use	ukb-b-12141	MR Egger	25.1748	53	0.9996
Antidepressant use	ukb-b-12141	IVW	31.2934	54	0.9943
Antidepressant use	ukb-b-873	MR Egger	9.5936	35	1.0000
Antidepressant use	ukb-b-873	IVW	13.0801	36	0.9998
Antidepressant use	ukb-b-3798	MR Egger	27.5196	45	0.9814
Antidepressant use	ukb-b-3798	IVW	28.3936	46	0.9808
Antidepressant use	ukb-b-9571	MR Egger	63.1418	65	0.5422
Antidepressant use	ukb-b-9571	IVW	82.6454	66	0.0809

Abbreviations: IVW, Inverse Variance Weighted; MR Egger, Mendelian Randomisation Egger.

## Discussion

This MR study provides robust genetic evidence of causal effects between antidepressant use and adverse bone health outcomes across multiple skeletal sites. By leveraging genome-wide association study data and utilising genetic variants as instrumental variables, our MR analysis helps mitigate several limitations inherent to observational epidemiology, including residual confounding and reverse causation. MR analysis approximates the design of randomised controlled trials by using genetic variation that is randomly assigned at conception [26]. Our comprehensive two-sample MR analysis identified statistically significant causal associations between genetically proxied antidepressant use and bone health deterioration, manifested through increased osteoporosis risk and fractures at multiple anatomical sites. While we did not apply formal multiple testing correction, the consistent positive associations across all five bone health outcomes, coupled with agreement across multiple MR methods, suggest that these findings are unlikely to be attributable to chance alone. Nevertheless, we acknowledge that some findings, particularly those with *p* values close to 0.05, should be interpreted with appropriate caution pending replication in independent cohorts.

In observational studies, individuals who use antidepressants are more likely to have other conditions that contribute to fracture risk, such as depression [27, 28, 29, 30]. Depression itself has been linked to reduced bone density and increased fracture risk, possibly due to physical inactivity [31, 32]. Additionally, antidepressant users in our study were more likely to have comorbid conditions such as chronic bronchitis, asthma, and a higher body mass index, all of which are recognised risk factors for osteoporosis [33, 34, 35, 36]. These factors may confound the relationship between antidepressant use and osteoporosis risk, potentially leading to an overestimation of the true effect in observational analyses.

The MR analysis provided evidence of a causal relationship between genetically proxied antidepressant use and an increased risk of wrist fractures. The IVW method indicated a statistically significant association, suggesting a potential effect of antidepressant use on wrist fracture risk. We identified significant causal associations with osteoporosis in two independent datasets, demonstrating consistency across complementary phenotypic definitions. These findings support a direct causal mechanism through which antidepressants influence bone mineral metabolism. The replication across two independent datasets strengthens confidence in the robustness of this finding, as discordant results across phenotypes would suggest potential population stratification or phenotypic heterogeneity [37]. Additionally, beyond osteoporosis, we observed significant causal associations with fractures at three anatomical locations—spine (OR = 1.0009, *p* = 0.0470), leg (OR = 1.0013, *p* = 0.0127), and wrist (OR = 1.0027, *p* = 0.0041)—indicating that antidepressant effects on fracture risk are not confined to a single skeletal site. Most notably, this site-specific variation is biologically meaningful and suggests differential vulnerability of distinct skeletal sites to antidepressant-mediated bone damage [17]. Furthermore, the marked site-specific variation in causal effects, with wrist fractures demonstrating the strongest association, provides intriguing insights into the pathophysiological mechanisms through which antidepressants influence bone health.

While wrist fractures demonstrated the strongest statistical association among the examined outcomes (OR = 1.0027, *p* = 0.0041), we emphasise that this represents a weak effect with an OR very close to unity. The relative risk increase of 0.27% per unit increase in genetically predicted antidepressant use, though statistically significant, translates to a modest absolute risk increase at the individual level. Therefore, these findings should be interpreted as suggestive evidence of a causal relationship requiring validation in independent cohorts and through complementary research designs, rather than definitive proof of clinically meaningful harm. Nevertheless, the site-specific pattern of associations, with the wrist showing relatively stronger effects compared to other skeletal sites, may provide insights into the mechanisms through which antidepressants influence bone health. The wrist is a common site for osteoporotic fractures, and factors affecting bone quality or fall risk could disproportionately impact this region [38]. The wrist is composed largely of high-turnover trabecular bone, making it especially sensitive to antidepressant-induced alterations in bone remodelling. Selective Serotonin Reuptake Inhibitors (SSRIs) and other serotonergic agents modulate serotonin-receptor signalling in osteoblasts and osteoclasts, potentially increasing bone resorption relative to formation, which may disproportionately affect the trabecular-rich distal radius [39]. Antidepressants can also cause orthostatic hypotension, dizziness, and impaired proprioception, increasing fall risk [40, 41, 42]. Because individuals instinctively extend their arms during a fall, the wrist becomes one of the most common fracture sites, and compromised bone quality further heightens fracture susceptibility. Fracture risk may not be fully explained by reductions in BMD. Antidepressants may alter collagen cross-linking, disrupt osteocyte function and microarchitecture, and increase microdamage, making bone more fragile even without critical BMD loss [43, 44]. The distal radius, which is rich in trabecular bone with relatively high turnover rates, may be particularly vulnerable to these qualitative deficits.

The spine’s mixed cortical-trabecular composition and load-bearing function make it a clinically important site for fracture prevention [45]. Even small increases in vertebral fracture risk have meaningful public health consequences in populations with high antidepressant use [20]. The causal effect on leg fractures reflects antidepressant effects on long bones, which are predominantly cortical in composition. The leg’s biomechanical importance for mobility and function underscores the clinical relevance of this finding. This result suggests that antidepressant effects are not limited to trabecular-rich sites but extend to cortical bone structures.

## Strengths and Limitations

This study has several strengths. The use of MR allowed us to assess causality while reducing confounding and reverse causation biases inherent in observational studies. However, there are limitations to consider. The outcome data were derived from GWAS summary statistics integrating large cohorts such as UK Biobank and FinnGen, where phenotype data were primarily collected through self-reporting and International Classification of Diseases (ICD) coding. Currently, large-scale GWAS data based on radiographically confirmed fractures are not publicly available. Importantly, in the MR framework, non-differential misclassification of outcomes typically biases effect estimates toward the null, reducing statistical power rather than generating false-positive results. Therefore, the significant associations observed in our study possess certain robustness, though true effects may be underestimated. Future validation using radiographically confirmed fracture outcomes is warranted when such data become available. In the MR analysis, using leg fracture data as a proxy for hip fractures may not fully capture the specific risk associated with hip fractures. Additionally, the population stratification could influence the MR results. The significant causal association found for wrist fractures in the MR analysis, while suggestive, is based on a small effect size and warrants cautious interpretation.

Additionally, an important limitation is that the exposure variable “antidepressant use” derived from GWAS data does not distinguish between antidepressant classes, dosages, treatment duration, or clinical indications. This limitation reflects an inherent constraint of currently available public GWAS datasets, which capture only binary information on antidepressant use without stratification by drug characteristics. As an exploratory study, our primary objective was to assess the broad causal relationship between general antidepressant use and bone health outcomes, providing preliminary evidence for subsequent refined investigations. We acknowledge this limitation may reduce the precision of mechanistic interpretation and limit the specificity of potential clinical implications. While our sensitivity analyses (MR-Egger intercept test, heterogeneity assessment, and leave-one-out analysis) showed no evidence of horizontal pleiotropy, we cannot completely exclude the possibility of residual pleiotropy. Additionally, our findings are based on individuals of European ancestry, and validation in populations of different ancestries is needed to assess generalisability. Finally, MR estimates reflect the causal effect of lifelong genetic predisposition to antidepressant use rather than the acute pharmacological effects of short-term treatment, which may differ in magnitude and clinical relevance.

## Future Research

Future studies should aim to validate these findings in longitudinal cohorts, using objective measures of bone density and fracture risk. Additionally, more comprehensive studies exploring the potential mechanisms by which antidepressants may influence bone health are warranted. When more granular GWAS data become available, future research should investigate the differential effects of specific antidepressant classes, dose-response relationships, and treatment duration on bone health outcomes. Such stratified analyses would enhance mechanistic understanding and provide more precise clinical guidance for antidepressant prescription in populations at risk for osteoporosis and fractures. Given the widespread use of antidepressants, particularly among older populations at higher risk of osteoporosis and fractures, understanding the potential impact of these medications on bone health remains a public health priority.

## Conclusion

MR evidence indicates that antidepressant use may exert a direct causal influence on bone metabolism and fracture susceptibility, with wrist fractures showing the most pronounced effect. These findings imply that antidepressant use may be associated with fracture risk at certain anatomical sites, particularly the wrist. Clinicians should exercise caution when prescribing antidepressants, especially to individuals at high risk of fractures, and consider bone health monitoring. Further research, particularly longitudinal studies and randomised controlled trials, is needed to clarify the relationship between antidepressant use and bone health, as well as to explore the mechanisms involved.

## Availability of Data and Materials

All GWAS summary statistics used in this study are publicly available through the IEU Open GWAS Project (https://gwas.mrcieu.ac.uk). The exposure data (antidepressant use, dataset ID: ebi-a-GCST90018998) and outcome data (osteoporosis: ukb-a-87, ukb-b-12141; fractures: ukb-b-873, ukb-b-3798, ukb-b-9571) can be accessed through the database. All R scripts and analytical code are available from the corresponding author upon reasonable request.
